# Seroprevalence of *Mycobacterium avium* subsp. *paratuberculosis* in Swiss dairy herds and risk factors for a positive herd status and within-herd prevalence

**DOI:** 10.3389/fvets.2024.1409694

**Published:** 2024-06-28

**Authors:** Martina Ottardi, Isabel Lechner, Jessica Wang, Sarah Schmitt, Marianne Schneeberger, Robin Michael Schmid, Roger Stephan, Mireille Meylan

**Affiliations:** ^1^Clinic for Ruminants, Vetsuisse Faculty, University of Bern, Bern, Switzerland; ^2^SAFOSO AG, Liebefeld, Switzerland; ^3^Institute for Food Safety and Hygiene, Section of Veterinary Bacteriology, Vetsuisse Faculty, University of Zürich, Zürich, Switzerland

**Keywords:** paratuberculosis, dairy, serology, ELISA, low seroprevalence, small herds, risk factors, Switzerland

## Abstract

**Introduction:**

Bovine paratuberculosis (PTB) is a chronic enteric disease caused by *Mycobacterium avium* subsp. *paratuberculosis* (MAP). Control of PTB is important given its negative economic consequences and the potential zoonotic role of MAP in Crohn’s disease in humans.

**Methods:**

To determine the seroprevalence of MAP in Swiss dairy herds and to identify risk factors associated with seropositive herd status and high within-herd seroprevalence, 10,063 serum samples collected from cattle over 12 months of age in 171 Swiss dairy farms were analyzed using a commercial ELISA test. Eight herds were excluded due to non-interpretable ELISA results. Risk factors associated with seropositive herd status and high within-herd seroprevalence were investigated with regression models using results from a questionnaire on management practices possibly associated with the introduction or spread of MAP in the remaining 163 herds. Univariable logistic regression was performed, carrying forward for multivariable regression analysis when *p* < 0.2.

**Results:**

The calculated between-herd true seroprevalence was 3.6% (95% CI, 0.96–8.4%). Due to the low within-herd seroprevalence, it was not possible to calculate the true seroprevalence at animal level; the apparent within-herd seroprevalence ranged from 2.3 to 5.5% with a median of 3.6% in nine positive farms. Herd size (*p* = 0.037) and the common grazing of lactating cows with cows from other herds (*p* = 0.014) were associated with seropositive herd status, while heifers sharing alpine pasture with dairy cattle from other herds were associated with a decreased probability of the herd to test seropositive (*p* = 0.042). Reliable identification of significant risk factors associated with MAP spread and high seroprevalence of PTB within seropositive herds was not possible due to low observed seroprevalence within herds and low sensitivity of the ELISA test.

**Discussion:**

These results highlight the limitation of serology for MAP diagnosis in small herds with low infection prevalence.

## 1 Introduction

Paratuberculosis (PTB) is a fatal chronic intestinal infection caused by *Mycobacterium avium* subsp. *paratuberculosis* (MAP), affecting primarily domestic and wild ruminants such as red deer, but also other wildlife including rabbits, foxes and badgers (1–4). Also known as Johne’s disease (JD), PTB is characterized by a long incubation period during which animals may remain subclinically infected for years (5). The cardinal clinical signs, weight loss and watery diarrhea, develop at a late stage of infection; finally, the disease leads to the death of affected animals. Young animals under 6 months of age are at the highest risk of becoming infected in herds with PTB (6). First reported in Germany at the end of the 19th century, PTB is now widely distributed throughout the world and is considered a significant disease due to its multiple negative impacts on economy and animal welfare (5, 7–9).

Intensive animal purchase, especially from multiple herds of origin, is a major risk factor associated with MAP introduction into cattle herds (10–13). Contact with feces of infected animals is also a potential risk factor for the introduction of the disease, and herd size is a recognized risk factor associated with a positive herd status as well (14, 15). Within-herd MAP transmission is primarily associated with contact of calves with adult cows’ feces (16), whereby transmission to young calves occurs mainly by ingestion of milk or feed contaminated with fecal material from infected animals (5). Beside indirect contamination of colostrum and milk through fecal material, MAP may also be directly secreted by the mammary gland of infected cows, especially in late stages of the disease (17–19). Intrauterine infection has also been described, particularly in cows with clinical JD (20). Management of the calving area and contact of the newborn calves with their dams are important points in the control of within-herd transmission (21, 22).

*Mycobacterium avium* subsp*. paratuberculosis* has been suspected to represent a zoonotic risk as a trigger agent for Crohn’s Disease (CD), a human chronic inflammatory bowel disease (23–29). Since MAP can be secreted directly into the milk of infected cows (18), a possible transmission way to humans could be through consumption of milk and dairy products (30). In many countries, cow milk is generally pasteurized prior to consumption, however, MAP has been shown to be able of surviving commercial pasteurization (31, 32). Insufficient understanding of MAP’s zoonotic potential and of its role in the development of human disease hampers a well-founded evaluation of the magnitude of its impact on public health (33, 34).

Economic losses due to PTB are mainly attributable to reduced milk production, animal replacement costs and decreased slaughter value (9, 35–39). In Switzerland, an annual economic loss of approximately CHF 4.6 millions due to reduced milk production was recently calculated for a population of 559,900 dairy cows (40). The fact that PTB can be spread through the movement of subclinically infected animals that contaminate their new environment, e.g., after purchase in a new herd, is considered to have contributed to the dissemination of JD (e.g., infected animals of continental European origin suspected of being a source of JD in Ireland), and has therefore serious implications for animal trade (9, 13, 41–44).

Although numerous countries have formal, mostly voluntary PTB control programs based on testing and culling strategies (9, 45, 46), these programs mostly have limited success due to the long incubation period and the low efficiency of diagnostic methods to identify infected animals during the subclinical phase of the disease (41, 47, 48). The sensitivity (Se) of different diagnostic tests varies considerably, ranging, e.g., between 7 and 94% for serum Enzyme-Linked Immunosorbent Assays (ELISA), 29 to 61% for milk ELISA, and 23 and 74% for fecal culture (FC), however, variations in study design and used diagnostic methods make comparison of different results almost impossible (49, 50). The delay of several years between infection time and detectable MAP shedding or immune reaction means that the same test is likely to perform better when used for animals with clinical JD than for animals in the subclinical stage of infection, e.g., the Se of a serum ELISA was found to be 87% in cows with clinical PTB and only 15% in subclinically infected animals (51–53). Likewise, serum ELISA showed a higher Se in animals with a heavy bacterial load (>50 colony forming units (CFU) per tube; Se = 75%) compared to low shedders (<10 CFU/tube; Se = 15%) (54). While FC allows for detecting infected animals earlier in the course of disease than other methods, it requires long incubation times (up to 16 weeks) until a definitive result is available (5, 52), and it can only detect animals excreting the organism, resulting in false-negative outcome in infected animals that have not yet started to shed the bacterium (55). Furthermore, the intensive laboratory work and expertise required for culturing are associated with high costs. In contrast, PCR methods are faster, easier and cheaper, but it has been shown that they may have a distinctly lower Se for MAP detection than FC (56). Therefore, despite the disadvantage of limited Se, quick and unexpensive serological tests are still commonly used to determine the prevalence of MAP infection at the herd level (49).

Numerous studies have been conducted worldwide to estimate PTB prevalence in cattle (9, 15, 46, 57, 58). More than 20% of the herds were considered to be infected with MAP in approximately half of 48 countries around the world for which data were available (9). The disease has been recently classified as enzootic in 27 European countries (including Switzerland), i.e., “countries where the disease was present and for which all periods of absence were shorter than 2 years” (46). Prevalence estimates have generally been higher for dairy cattle than for beef cattle (7). In North America, a study conducted in the 17 main dairy U.S. states in 2007 indicated that 68.1% herds had at least one cow that tested positive on FC (59). In Europe, the apparent between-herd seroprevalence in cattle was reported to range from 38 to 68% (60). However, available prevalence studies are heterogeneous and it is difficult to compare their results due to differences in sampling design, diagnostic strategies and case definitions (41). In many regions of the world, the prevalence of PTB is still completely unknown (45).

In Switzerland, where PTB is a notifiable disease, approximately 60 bovine cases have been reported yearly in the last 3 years (61), however, given the long subclinical phase of infection and unspecific clinical signs, PTB may be more common but affected animals are culled without diagnosis confirmation. Sparse prevalence data at the herd and at the animal level are also available from earlier studies (62–66), but current information about the actual prevalence of PTB in Swiss dairy herds is not available, despite the importance of the dairy industry in Switzerland. The aim of the present study was to determine the between-herd and the within-herd seroprevalence of PTB in a representative subset of Swiss dairy herds. A serum ELISA method was used to allow for inclusion of large numbers of animals and herds. In addition, information about the herds was collected in order to investigate risk factors associated with positive herd status and seroprevalence within positive herds.

## 2 Materials and methods

### 2.1 Study design

Farms were recruited for an observational seroprevalence study and risk factor analysis on PTB in a representative subset of the Swiss dairy population.

### 2.2 Sample size

The numbers of herds and animals to be sampled was calculated in a two-step procedure using Epitools® (67), based on the data available from previous studies in Switzerland (62, 64, 68, 69). First, the sample size to assess the within-herd prevalence was determined, whereby a minimum Se and Sp of ≥95% for herd status determination was set as target performance. A within-herd prevalence of 20% and a median of 30 animals ≥1 year old were assumed for the calculation. The diagnostic test Se and Sp were 58.2 and 99.0%, based on test characteristics of an approved ELISA tests (IDEXX Paratuberculosis Screening Ab Test, IDEXX Montpellier SAS, Montpellier, France) by the Friedrich-Loeffler-Institute (70). Second, the sample size to assess the between-herd prevalence was determined, assuming a between-herd prevalence of 20% and using the target Se and Sp of the within-herd calculations. This resulted in a target sample size of 300 herds (confidence level of 95%, precision of 5%).

### 2.3 Study population: farms and animals

“Herd size” was defined as the number of dairy cows (in lactation and dry cows), and “number of animals” was used to describe the total of animals tested including heifers from the age of 12 months until 2 years throughout the manuscript. Inclusion criteria for the farms were a herd size of at least 25 dairy cows (in order to achieve the calculated 30 tested animals when including cows and heifers over the age of 12 months) and membership in at least one of the main Swiss dairy breeding associations: Holstein, Braunvieh and Swissherdbook, which represented about 88% of the registered dairy cows in Switzerland in 2022 (71–73). Heifers between the age of 12 and 24 months were included in the study population based on the results of a previous Swiss study (66) in which 3.9% of the heifers in this age category had been found to be shedding MAP (positive fecal culture), in 6 out of 13 participating herds. Participants for the study were recruited via an email sent by the breeding associations. The email comprised a document describing the project and a link for online enrolment through a short questionnaire (SurveyMonkey platform, Momentive Global Inc., Delaware St, San Mateo, USA). The questionnaire, available in German, French and Italian, consisted of 19 questions, the first five ones about the farmers’ contact information and identification number of the farm (in the Swiss animal movement database, TVD), and the remaining ones about farm management and animals (type of production, average number of animals, main breed, use of alpine pastures and whether young animals went to heifer raising facilities). The responding farmers gave written informed consent for access to individual cow data (cow identification, date of birth, last date of calving) by the study team as well as the farm’s membership number in one (or more) breeding association(s) to obtain information on milk yield and quality as well as on reproduction parameters. The email was sent to approximately 11,000 possible participants fulfilling the inclusion criteria in all regions of Switzerland. The survey remained open for 3 months from July 19, 2021, to September 21, 2021. The study was approved by the competent Veterinary authorities (animal experimentation authorization number BE 32–2021).

### 2.4 Sampling

Each farmer who had enrolled in the study via the online questionnaire was contacted by telephone to confirm his/her willingness to participate in the study and to set up an appointment for a farm visit. Farm visits took place between November 2021 and October 2022, each farm was visited once. Farmers who reported sending (mostly young) animals to alpine pastures during the summer were visited during the winter period to gain access to all testable animals. All cows (in lactation and dry) and heifers older than 12 months and less than 2 years were included in the study. Blood samples were collected from the coccygeal vein using Serum Monovettes^®^ (9 mL Z, SARSTEDT AG & Co. KG, Nümbrecht, Germany). The samples were transported in a container refrigerated at 1°C to the Vetsuisse Faculty Bern, where they were centrifuged (2,123 *g*, 10 min) and the serum separated within maximal 12 h of collection. The serum was transferred to 2 mL Micro Tubes^®^ (SARSTEDT AG & Co. KG, Nümbrecht, Germany) and frozen at −20°C. The frozen serum samples were then transported at monthly intervals to the Institute of Veterinary Bacteriology of the University of Zurich for analysis.

### 2.5 Serological analysis

The frozen sera were thawed and tested with the commercial ELISA ID Screen Paratuberculosis Indirect Screening Test [IDvet, Grabels, France; Se 58.2%, Sp 99.3% (70)] according to the manufacturer’s instructions. The sera were tested in duplicate, and the optical densities (OD) were recorded at 450 nm. Results were interpreted according to the manufacturer’s instructions (greater than or equal to 70% was considered positive, less than or equal to 60% was considered negative, and greater than 60% and less than 70% was considered doubtful).

### 2.6 Farm questionnaire

The questionnaire to assess risk factors for MAP introduction and spread in the farms was developed in Microsoft Access 2016 (Microsoft Corporation, Redmond, Washington, USA). Questions were based on questionnaires used in previous studies (13, 16, 48, 66, 74–78), the questionnaire was validated with 3 farmers prior to use in the frame of the study. The questionnaire was filled at the end of the farm visit, after blood sampling, during a personal interview with the farm manager. If the manager was not present at that time, the interview was done later by phone. Information on farm demographics and management was gathered with the questionnaire (Table 1), as well as information about 10 major themes including calving area, housing, feeding management, manure and slurry management, use of pasture and/or alpine pasture, animal trade, knowledge of PTB, previous cases of the disease, and a last section about hygiene (Tables 2–5). The animals on the farm were assigned to 6 categories according to their age (neonates in the first 2–3 weeks of life (housed in individual hutches or small groups), older (pre-weaned) calves until weaning, post-weaned calves until 1 year of age, bred heifers from approximately 1 year until calving, lactating cows, and dry cows) according to the handbook for the Risk Assessments and Management Plans for Johne’s Disease of the National Johne’s Working Group (41). The questionnaire sections on housing, feeding, pasture and alpine pasture were recorded for each age group separately.

**Table 1:** Summary of farm demographics, herds characteristics and previous experience with paratuberculosis (PTB) for the 163 Swiss dairy farms participating in the study, classified as seropositive (*n* = 9) or seronegative (*n* = 154) for PTB based on serum ELISA results.

	Total	Seropositive farms	Seronegative farms
Farm size (hectares)			
Median	37	45	35
IQR^1^	25.0–50.0	30.0–75.0	25.0–50.0
Range (minimum-maximum)	11–160	25–85	11–160
Herd size (all cows ≥2 years)			
Median	42	50	41
IQR^1^	32–57	41–55	32–57
Range	16–138	36–138	16–133
Average milk yield (liters/cow/year)			
Median	7,800	8,600	7,800
IQR^1^	7,000–9,000	7,500–9,000	7,000–9,000
Range	5,000–17,000	5,500–17,000	5,000–15,000

	Total (%)	Seropositive farms (%)	Seronegative farms (%)
Farm PTB-status	163 (100)	9 (5.5)	154 (94.5)
Agricultural zone			
Midland zone	86 (52.7)	4 (44.4)	82 (53.2)
Hill zone	25 (15.3)	3 (33.3)	22 (14.3)
Mountain zones^2^	52 (32.0)	2 (22.2)	50 (32.5)
Stall system			
Free stall	140 (85.9)	8 (88.9)	132 (85.7)
Tie-stall	23 (14.1)	1 (11.1)	22 (14.3)
Production system			
Conventional^3^	91 (55.8)	6 (66.7)	85 (55.2)
Label^4^	55 (33.7)	3 (33.3)	52 (33.8)
Organic^5^	17 (10.5)	0 (0)	17 (11.0)
Reproduction management			
Breading bull on farm	37 (22.7)	1 (11.1)	36 (23.4)
Artificial insemination only	126 (77.3)	8 (88.9)	118 (76.6)
Main cattle breed			
Holstein/Red Holstein	78 (47.9)	6 (66.7)	72 (46.8)
Brown Swiss	49 (30.0)	1 (11.1)	48 (31.2)
Swiss Fleckvieh	15 (9.2)	0 (0)	15 (9.7)
Other breeds^6^	21 (12.9)	2 (22.2)	19 (12.3)
Seasonal calving^7^			
Yes	11 (6.7)	2 (22.2)	9 (5.8)
No	152 (93.3)	7 (77.8)	145 (94.2)
Previous PTB cases in the herd			
Yes	13 (8.0)	5 (55.6)	8 (5.2)
No	122 (74.8)	3 (33.3)	119 (77.3)
Unknown	28 (17.2)	1 (11.1)	27 (17.6)
Origin of the sick animals			
Animal(s) raised on farm	11 (84.6)	4 (80.0)	7 (87.5)
Purchased animal(s)	2 (15.4)	1 (20.0)	1 (12.5)

^1^Interquartile range: first quartile (25th percentile) – third quartile (75th percentile).

^2^Including all four mountain zones defined in the Swiss agricultural system: map.geo.admin.ch.

^3^As described in: Ökologischer Leistungsnachweis (admin.ch).

^4^As described in: www.ipsuisse.ch.

^5^As described in: www.biosuisse.ch.

^6^Including Jersey, Montbéliarde, Kiwi-Cross, Normande, Simmentaler and crossbreeds.

^7^Seasonal calving: systems focused on having most cows calve over a short period (about 12 weeks) starting in late winter so that the period of maximum feed demand coincides with the period of peak pasture growth rates around mid-spring.

**Table 2:** Herd level occurrence of characteristics and management practices included in the analyses of risk factors potentially associated with a seropositive herd status for paratuberculosis in Swiss dairy herds, for all participating farms (total, *n* = 163) and for serologically positive (*n* = 9) and negative (*n* = 154) herds separately.

	Total (%)	Seropositive farms (%)	Seronegative farms (%)
Type(s) of production on the farm			
Exclusively dairy	121 (74.2)	6 (66.7)	115 (74.7)
Dairy and fattening of calves born on the farm as veal and/or beef	35 (21.5)	2 (22.2)	33 (21.4)
Dairy and fattening of purchased veal calves	4 (2.5)	0 (0)	4 (2.6)
Dairy and fattening of purchased beef calves	3 (1.8)	1 (11.1)	2 (1.3)
Presence of a bull in the herd			
No bull or one raised on the farm	115 (70.6)	7 (77.8)	108 (70.0)
New bull purchased <1x per year	23 (14.1)	0 (0)	23 (15.0)
New bull purchased every year	25 (15.3)	2 (22.2)	23 (15.0)
Origin of the farm’s water supply			
Communal water source only	95 (58.3)	5 (55.6)	90 (58.4)
Private well (with or without additional communal water source)	68 (41.7)	4 (44.4)	64 (41.6)
Contact with animals from other herds during the grazing period^8^			
Calves (pre-weaned and post-weaned)			
No^9^	163 (100)	9 (100)	154 (100)
Heifers			
No^9^	103 (63.2)	6 (66.7)	97 (63.0)
With youngstock^10^	59 (36.2)	3 (33.3)	56 (36.4)
With adult cattle^11^	1 (0.6)	0 (0)	1 (0.6)
Lactating cows			
No^9^	160 (98.0)	8 (88.9)	152 (98.7)
With adult cattle^11^	3 (2.0)	1 (11.1)	2 (1.3)
Dry cows			
No^9^	157 (96.3)	8 (88.9)	149 (96.8)
With adult cattle^11^	6 (3.7)	1 (11.1)	5 (3.2)
Shared alpine pasture with animals from other herds^12^			
Pre-weaned calves			
No^13^	161 (98.7)	9 (100)	152 (98.7)
With adult cattle^11^	2 (1.3)	0 (0)	2 (1.3)
Post-weaned calves			
No^13^	140 (85.9)	9 (100)	131 (85.1)
With youngstock^10^	16 (9.8)	0 (0)	16 (10.3)
With adult cattle^11^	7 (4.3)	0 (0)	7 (4.6)
Heifers			
No^13^	28 (17.2)	4 (44.4)	24 (15.6)
With youngstock^10^	110 (67.5)	5 (55.6)	105 (68.2)
With adult cattle^11^	25 (15.3)	0 (0)	25 (16.2)
Lactating cows			
No^13^	134 (82.2)	8 (88.9)	126 (81.8)
Yes^14^	29 (17.8)	1 (11.1)	28 (18.2)
Dry cows			
No^13^	141 (86.5)	9 (100)	132 (85.7)
Yes^14^	22 (13.5)	0 (0)	22 (14.3)
Participation to cattle shows (≥once a year)			
Yes	62 (38.0)	3 (33.3)	59 (38.3)
No	101 (62.0)	6 (66.7)	95 (61.7)
Purchase of breeding animals			
No	109 (66.9)	6 (66.7)	103 (66.9)
Only as young animals^10^	5 (3.0)	0 (0)	5 (3.2)
At least one adult animal^15^ per year	49 (30.1)	3 (33.3)	46 (29.9)
Number of source farms for purchase^16^			
No purchase	77 (47.2)	2 (22.2)	75 (48.7)
From one farm	31 (19.0)	2 (22.2)	29 (18.8)
From > one farm	55 (33.8)	5 (55.6)	50 (32.5)
Information request about the source farm^17^			
Yes	9 (5.5)	1 (11.1)	8 (5.2)
No	154 (94.5)	8 (88.9)	146 (94.8)
Heifers raised on a rearing farm^18^			
Yes	56 (34.4)	2 (22.2)	54 (35.1)
No	107 (65.6)	7 (77.8)	100 (64.9)

^8^Direct contact of the different animal categories with animals from other farms during the grazing period.

^9^No direct contact with animals from other farms or only via adjacent pastures.

^10^Including calves and heifers.

^11^Including lactating and/or dry cows.

^12^Shared alpine summer pasture with animals from other farms for the different animal categories.

^13^Animals of the farm do not go to summer pasture or they do not share it with animals from other farms.

^14^Including any combination of calves, heifers and cows.

^15^Including cows or bulls.

^16^Number of farms from which cattle was purchased in the last year.

^17^Request for information on potential diseases in the farm of origin by the farmer prior to purchase.

^18^Young animals raised in an external rearing farm.

**Table 3:** Herd level occurrence of characteristics and management practices potentially associated with contact between animals of different age categories within a farm included in the analysis of risk factors potentially associated with the prevalence level within herds seropositive for paratuberculosis in Swiss dairy herds, for all participating farms (total, *n* = 163) and for serologically positive (*n* = 9) and negative (*n* = 154) herds separately.

	Total (%)	Seropositive farms (%)	Seronegative farms (%)
Presence of other ruminants on the farm^19^			
Yes	55 (33.7)	3 (33.3)	52 (33.8)
No	108 (66.3)	6 (66.7)	102 (66.2)
Presence of other food producing animals (i.e., pigs)^20^ on the farm			
No	129 (79.1)	8 (88.9)	121 (78.6)
<20 pigs	12 (7.4)	0 (0)	12 (7.8)
≥20 pigs	22 (13.5)	1 (11.1)	21 (13.6)
Calving management			
Contact with other cows during calving			
No, only individual calving^21^	75 (46.0)	4 (44.5)	71 (46.1)
Low contact intensity^22^	26 (16.0)	1 (11.0)	25 (16.2)
Intensive contact (group calving box)^23^	62 (38.0)	4 (44.5)	58 (37.7)
Bedding management for calving^24^			
Change of bedding after each calving	28 (17.2)	3 (33.3)	25 (16.2)
Pen or stall freshly bedded before each calving^25^	122 (74.8)	4 (44.5)	118 (76.6)
Same bedding used several times^26^	13 (8.0)	2 (22.2)	11 (7.2)
Gloves for the care of newborn calves			
Yes	6 (3.7)	0 (0)	6 (3.9)
No	157 (96.3)	9 (100)	148 (96.1)
Use of the calving pen as sick pen			
Never	20 (12.3)	1 (11.1)	19 (12.3)
Rarely	34 (20.6)	2 (22.2)	32 (20.8)
Often	16 (9.8)	0 (0)	16 (10.4)
Always	71 (43.5)	4 (44.5)	67 (43.5)
N/A^27^	22 (13.5)	2 (22.2)	20 (13.0)
Colostrum from the dam’s udder^28^			
Yes	80 (49.0)	2 (22.2)	78 (50.6)
No	83 (51.0)	7 (77.8)	76 (49.3)
Housing of the young animals			
Neonates (first 2–3 weeks of life)			
Individual hutch^29^	92 (56.4)	5 (55.5)	87 (56.5)
Group pen	71 (43.6)	4 (44.5)	67 (43.5)
Pre-weaned calves			
Individual hutch^29^	3 (1.8)	0 (0)	3 (1.9)
Group pen	156 (95.7)	9 (100)	147 (95.5)
Not on the farm (rearing farm)^18^	4 (2.5)	0 (0)	4 (2.6)
Post-weaned calves			
Group pen	137 (84.0)	7 (77.8)	130 (84.4)
Not on the farm (rearing farm)^18^	26 (16.0)	2 (22.2)	24 (15.6)
Risk of fecal contamination from the cows to the calves’ environment			
Neonates (first 2–3 weeks of life)			
No^30^	4 (2.5)	1 (11.1)	3 (1.9)
Low^31^	151 (92.6)	7 (77.8)	144 (93.5)
Moderate^32^	0 (0)	0 (0)	0 (0)
High^33^	8 (4.9)	1 (11.1)	7 (4.6)
Pre-weaned calves			
No^30^	6 (3.7)	1 (11.1)	5 (3.3)
Low^31^	147 (90.2)	7 (77.8)	140 (90.9)
Moderate^32^	3 (1.8)	0 (0)	3 (1.9)
High^33^	7 (4.3)	1 (11.1)	6 (3.9)
Post-weaned calves			
No^30^	14 (8.6)	0 (0)	14 (9.1)
Low^31^	32 (19.6)	2 (22.2)	30 (19.5)
Moderate^32^	111 (68.1)	7 (77.8)	104 (67.5)
High^33^	6 (3.7)	0 (0)	6 (3.9)
Shared alpine pastures with other animal categories of the farm			
Heifers			
No	134 (82.2)	9 (100)	125 (81.2)
Yes^34^	29 (17.8)	0 (0)	29 (18.8)
Lactating cows			
No^35^	156 (95.7)	9 (100)	147 (95.5)
Yes^34^	7 (4.3)	0 (0)	7 (4.5)
Dry cows			
No^35^	148 (90.8)	9 (100)	139 (90.3)
Yes^34^	15 (9.8)	0 (0)	15 (9.7)

^19^Including goats, sheep, new world camelids, water buffalo, deer.

^20^No food producing animals other than pigs were present in the participating farms.

^21^Including individual calving pen or tie stall where the neighboring cows are removed prior to a calving.

^22^Calving on pasture (extensive) or tie stall where the neighboring cows are not removed before calving (maximal two cows).

^23^Calving in the barn or group calving pen (up to 20 cows).

^24^Cleaning of calving pen and for tie stall change of bedding before the calving.

^25^Bedding not removed, new clean material added.

^26^In case of tie stall: not freshly bedded before calving.

^27^N/A: not applicable (no calving pen).

^28^Colostrum intake through direct nursing from the dam.

^29^Single housing in box or igloo, only one calf per box or igloo with visual contact with the other calves but no physical contact.

^30^No contamination possible on the farm, or the calves are not on the farm (rearing farm).

^31^Low risk: indirect contamination through the use of same cleaning or feeding equipment and boots both for the calves’ and the cows’ area.

^32^Moderate risk: calves are grazed on the same pastures or alp pasture with cows.

^33^High risk: calves are housed with the cows or can come in contact with cows or cow manure in their housing.

^34^All combinations of animals of different ages from the same farm.

^35^Not with young animals but possible contact between lactating and dry cows.

**Table 4:** Herd level hygiene management practices potentially associated with the spread of *M. avium* subsp. *paratuberculosis* within a farm included in the analysis of risk factors potentially associated with the prevalence level within herds seropositive for paratuberculosis in Swiss dairy herds, for all participating farms (total, *n* = 163) and for serologically positive (*n* = 9) and negative (*n* = 154) herds separately.

	Total (%)	Seropositive farms (%)	Seronegative farms (%)
Frequency of manure removal			
Neonates (first 2–3 weeks of life)			
1-2x/day	24 (14.7)	0 (0)	24 (15.6)
<1x/day	139 (85.7)	9 (100)	130 (84.4)
Pre-weaned calves			
>3x/day	3 (1.8)	1 (11.1)	2 (1.3)
1-2x/day	26 (16.0)	0 (0)	26 (16.9)
<1x/day	130 (79.7)	8 (88.9)	122 (79.2)
N/A^36^	4 (2.5)	0 (0)	4 (2.6)
Post-weaned calves			
>3x/day	12 (7.4)	1 (11.1)	11 (7.1)
1-2x/day	48 (29.4)	2 (22.2)	46 (29.9)
<1x/day	77 (47.2)	4 (44.5)	73 (47.4)
N/A^36^	26 (16.0)	2 (22.2)	24 (15.6)
Heifers			
>3x/day	22 (13.5)	1 (11.1)	21 (13.6)
1-2x/day	45 (27.6)	3 (33.3)	42 (27.3)
<1x/day	48 (29.4)	3 (33.3)	45 (29.2)
N/A^36^	48 (29.4)	2 (22.3)	46 (29.9)
Lactating cows			
>3x/day	109 (66.9)	5 (55.6)	104 (67.5)
1-2x/day	42 (25.8)	4 (44.4)	38 (24.7)
<1x/day	12 (7.3)	0 (0)	12 (7.8)
Dry cows			
>3x/day	67 (41.1)	3 (33.3)	64 (41.6)
1-2x/day	59 (36.2)	4 (44.5)	55 (35.7)
Not daily	37 (22.7)	2 (22.2)	35 (22.7)
Cleaning management in the calf area			
Neonates (first 2–3 weeks of life)			
Regular^37^ disinfection^38^	16 (9.8)	1 (11.1)	15 (9.7)
1-4x/year disinfection^39^	79 (48.5)	5 (55.6)	74 (48.1)
Cleaning/washing^40^	11 (6.7)	0 (0)	11 (7.1)
No disinfection or washing	57 (35.0)	3 (33.3)	54 (35.1)
Pre-weaned calves			
Regular^37^ disinfection^38^	9 (5.5)	1 (11.1)	8 (5.2)
Disinfection 1-4x/year^39^	26 (16.0)	1 (11.1)	25 (16.2)
Cleaning/washing^40^	2 (1.2)	0 (0)	2 (1.3)
No disinfection or washing	126 (77.3)	7 (77.8)	119 (77.3)
Post-weaned calves			
Regular^37^ disinfection^38^	1 (0.6)	0 (0)	1 (0.6)
Disinfection 1-4x/year ^39^	9 (5.5)	0 (0)	9 (5.8)
Cleaning/washing^40^	1 (0.6)	0 (0)	1 (0.6)
No disinfection or washing	152 (93.3)	9 (100)	143 (93.0)
Runoff from the manure pile^41^			
Yes	114 (70.0)	8 (88.9)	106 (68.8)
No	49 (30.0)	1 (11.1)	48 (31.2)
Use of manure equipment for other tasks^42^			
No	158 (96.9)	9 (100)	149 (96.8)
After cleaning also for feed^43^	5 (3.1)	0 (0)	5 (3.2)
Occurrence of diseases at the herd level at the time of farm visit^44^			
Calves			
Yes	119 (73.0)	1 (11.1)	118 (76.6)
No	40 (24.5)	8 (88.9)	32 (20.8)
N/A^36^	4 (2.5)	0 (0)	4 (2.6)
Heifers			
Yes	105 (64.4)	6 (66.7)	99 (64.3)
No	6 (3.7)	1 (11.1)	5 (3.2)
N/A^36^	52 (31.9)	2 (22.2)	50 (32.5)
Cows^45^			
Yes	49 (30.0)	0 (0)	49 (31.8)
No	114 (70.0)	9 (100)	105 (68.2)

^36^N/A: not applicable (youngstock in external rearing farm).

^37^ Regular: after each group.

^38^ Disinfection with a product active against mycobacteria (i.e., Neopredisan^®^ or Noviralx3^®^; other disinfectants without efficacy against mycobacteria used in six farms are listed under “cleaning/washing”).

^39^Disinfection of the area > 1x year.

^40^Area cleaned with hot water, high pressure or disinfected with a disinfectant without efficacy against mycobacteria.

^41^Contamination of the environment around the manure pile.

^42^Vehicles and equipment for loading and transporting manure also used for other purposes (feed transport).

^43^Cleaned with water.

^44^Presence of diseases considered as a problem at the herd level at the time of the visit, including: for calves digestive, respiratory, or umbilical diseases; for heifers digestive diseases and fertility problems; for cows digestive diseases, fertility problems and poor udder health; or any other problems mentioned by the herd managers for every category.

^45^Including lactating and dry cows.

**Table 5:** Herd level feeding management practices potentially associated with the spread of *M. avium* subsp. *paratuberculosis* within a farm included in the analysis of risk factors potentially associated with the prevalence level within herds seropositive for paratuberculosis in Swiss dairy herds, for all participating farms (total, *n* = 163) and for serologically positive (*n* = 9) and negative (*n* = 154) herds separately.

	Total (%)	Seropositive farms (%)	Seronegative farms (%)
Feeding management			
Milk feeding^46^			
Powdered milk	13 (8.0)	2 (22.2)	11 (7.1)
Fresh milk (with/without milk powder)^47^	150 (92.0)	7 (77.8)	143 (92.9)
Leftovers from the cows’ feed given to post-weaned calves			
Yes	15 (9.2)	0 (0)	15 (9.7)
No	148 (90.8)	9 (100)	139 (90.3)
Possible fecal contamination of calf feed^48^			
Yes	10 (6.1)	0 (0)	10 (6.5)
No	153 (93.9)	9 (100)	144 (93.5)
Leftovers from the cows’ feed given to heifers			
Yes	34 (20.9)	2 (22.2)	32 (20.8)
No	129 (79.1)	7 (77.8)	122 (79.2)
Possible water contamination^49^ from cows to calves (all calf categories)			
Yes	3 (1.9)	0 (0)	3 (1.9)
No	160 (98.1)	9 (100)	151 (98.1)
Use of manure and/or slurry for fertilization			
For crop land and/or hay meadows	5 (3.0)	0 (0)	5 (3.2)
For cow and heifer pastures	44 (27.0)	2 (22.2)	42 (27.3)
For pastures, including calf pastures	114 (70.0)	7 (77.8)	107 (69.5)

^46^Feeding of the calves after colostrum administration.

^47^Milk or a mix of milk and milk powder.

^48^Possible contamination of calves’ feed with cow manure in the feed storage or on the feeding place.

^49^Possible fecal contamination of the calves’ water with cow manure.

### 2.7 Data management and statistical analyses

Data from the risk assessment questionnaire were recorded in a Microsoft Excel file (Microsoft Corporation, Redmond, Washington, USA). Additional farm production data made available by the breeding associations included farm annual production reports, individual monthly milk weighing and standard lactation data, as well as insemination and calving dates of the cows. Individual cow data were provided directly by the participants as Microsoft Excel files or obtained through the Swiss online counter for agriculture (79). The participating herds were classified as positive or negative for PTB based on ELISA results. Since farms of different sizes were included, the cut-off for a seropositive herd status was adjusted so that both Se and Sp remained above 95%: one serologically positive animal defined the herd as seropositive if herd size (number of animals aged 2 years or more years, i.e., cows in lactation and dry cows) was 38 or less, two positives for 39–81 animals, and three for 85–138 animals. The number of reactors was assessed with the Herd Sensitivity/Specificity Calculator of Scotland’s Rural College [SRUC (80)]. In addition to the apparent seroprevalence, the true seroprevalence accounting for the diagnostic test characteristics was calculated, for within-herd prevalence using the diagnostic test characteristics and for between-herd prevalence using the median Se and S*p* values over all herds; these calculations were performed with Epitools^®^ (67).

A risk factor analysis was performed to identify possible associations of management factors with a seropositive herd status as well as with the within-herd prevalence in infected herds. For the identification of possible associations of management factors with a seropositive herd status, factors possibly associated with MAP introduction in dairy herds were assessed (Table 2). For the risk factor analysis considering the within-herd prevalence, risk factors influencing MAP spread within infected herds were considered (Tables 3–5). Since youngstock of some herds were raised in external rearing farms, and because of differences due to barn types (free stall vs. tie stall), some answers were missing for questions about young animals and calving management. To minimize missing data, some of the original questionnaire variables and their categories were grouped for analyses (see Supplementary Tables S1, S2). The data for risk factors possibly associated with a seropositive herd status were analyzed in two categories (Supplementary Table S1).

Statistical analyses were performed using the R software, version 4.2.3 (R Core Team 2023). Before conducting the main analyses, the number of cows, which was used as a proxy for herd size, was logarithmized. Moreover, pairwise correlation tests to the response variables were applied to prevent multicollinearity and model overfitting. Spearman’s rank correlation matrix was performed and any pair of variables with a correlation coefficient (|r|) greater than 0.8 was considered highly correlated (81). However, no variable was collinear, and, therefore, all response variables were retained for further analysis. For both datasets, an univariable logistic regression was performed, carrying forward for multivariable regression analysis when *p* < 0.2. Regarding the multivariable regression analysis, variables were removed if the constructed model exhibited the lowest Akaike information criterion (AIC) after using the stepwise elimination method, aiming at achieving the most parsimonious model. The multivariable regression models set a significance cut-off of *p* < 0.05. For the first model analysis, the response variables were farms seropositive for PTB according to the definition given above. After selecting the most parsimonious model in the multivariable model, interactions among the response variables were assessed. For the second model, the response variable was the prevalence of PTB seropositivity (number of seropositive cases/number of tested animals *100) in each herd. Lastly, all resulting models were inspected visually for homoscedasticity and normality.

## 3 Results

### 3.1 Study population: farms and animals

Serum samples were collected in 171 dairy farms distributed over all 26 cantons of Switzerland, except for Appenzell Inner-Rhodes, Basel-City, Geneva and Obwalden. Median herd size was 42 dairy cows aged 2 years or more (IQR: 32–57), with Holstein being the predominant breed (in 47.9% of all farms, 66.7% of positive farms and 54.9% of seropositive animals). A total of 10,063 animals (7,943 cows, 2,091 heifers and 29 bulls) were tested for antibodies against MAP, the number of animals tested per herd ranged from 22 to 199, with a median of 8 heifers aged between 1 and 2 years and 42 cows aged 2 years or more. A case of PTB was reported to have previously occurred in 13 farms (8%). Participation to the study was on a voluntary basis and recruiting 300 farms with a minimum of 30 animals for testing turned out to be impossible. Therefore, slightly smaller farms with a minimum of 25 animals were also included in the study. In three cases, however, less than 25 animals were eventually available for testing (once 22 and twice 23, respectively) because the heifers were not on site at the time of sampling. Thus, the target sample size of 300 herds could not be achieved. However, since several very large (under Swiss dairying conditions) farms were sampled, the average herd-level Se was higher than the initially set target of 95%. Consequently, a reasonable CI for the final between-herd prevalence calculations could be achieved despite the lower than expected number of participating herds.

### 3.2 Serological analyses

Of 10,063 serum samples that were tested for antibodies against MAP, 51 (46 cows and 5 heifers) were positive, 10,000 (7,886 cows, 2,085 heifers and 29 bulls) were negative, and 12 (11 cows and one heifer) were doubtful. Eight farms (with 453 animals, 347 cows and 106 heifers) had to be excluded from further analyses because one (or several) ELISA result in the herd was doubtful and the farm could not be reliably classified as negative or positive. The ELISA results of the remaining 163 herds are presented in Table 6. Fifty-one of the 9′610 remaining samples (0.53%) tested positive, most of them were cows (*n* = 46/7,596, 0.61%) but five heifers (between the age of one and 2 years) were also antibody positive (*n* = 5/1,985, 0.25%); all tested bulls were seronegative. Information on farm demographics and herd characteristics is shown in Table 1.

**Table 6:** Results of the serum ELISA test (ID Screen^®^ Paratuberculosis Indirect Screening Test, IDvet, Grabels, France) for the detection of antibodies against *Mycobacterium avium* subsp. *paratuberculosis* in 9,610 bovines aged 1 year or more in 163 Swiss dairy herds.

Results	*n* cows	*n* heifers >1 year	*n* bulls	Total (%)
Positive	46	5	0	51 (0.53)
Negative	7,550	1,980	29	9,559 (99.47)
Total	7,596	1,985	29	9,610 (100)

**Figure 1:**
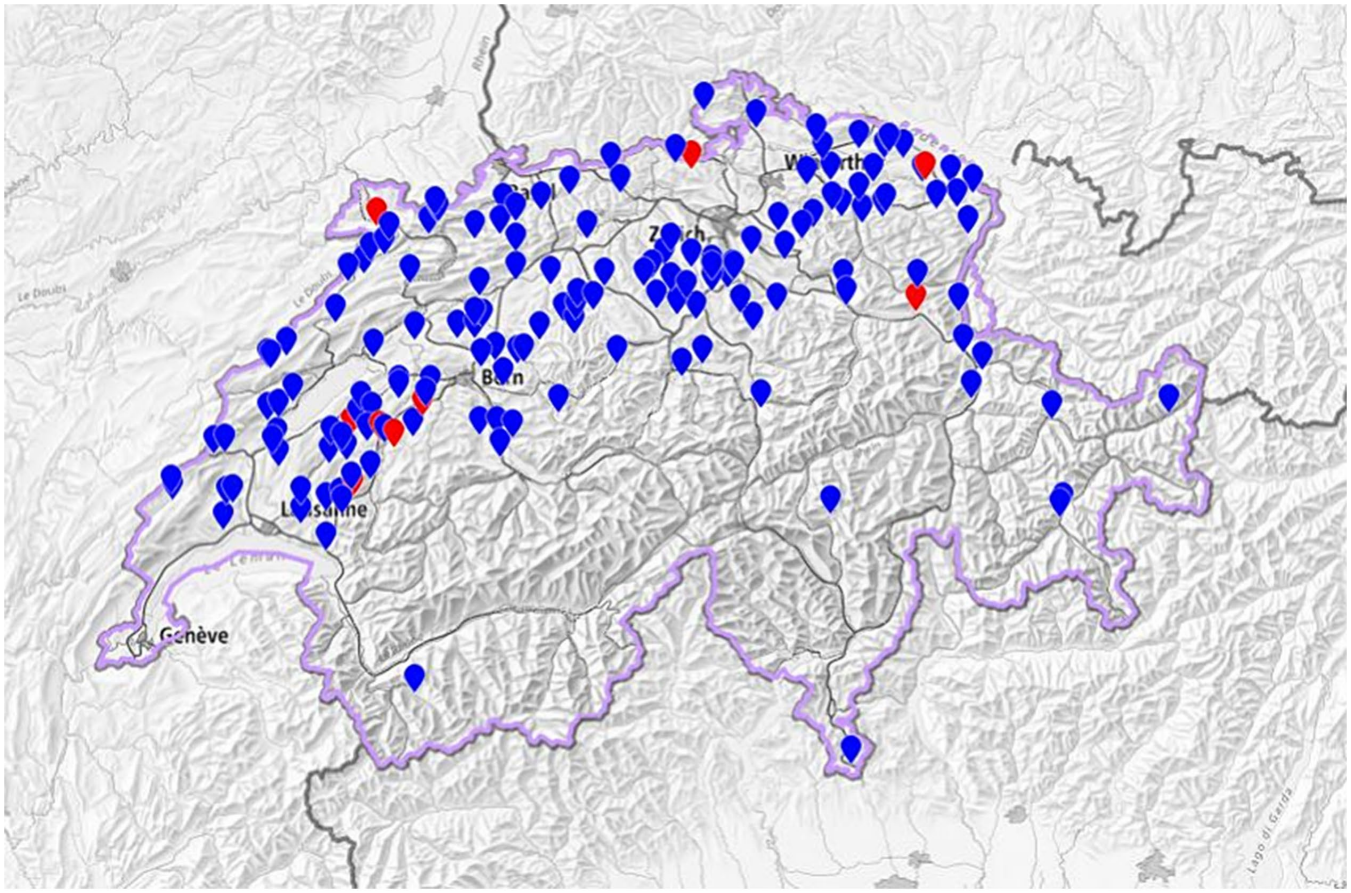
Geographical distribution of the 163 Swiss dairy farms included in the study [9 seropositive (red dots) and 154 seronegative (blue dots) herds]. Based on study data mapped in the Swiss Federal Geoportal https://map.geo.admin.ch/.

At the herd level, nine of the remaining 163 farms (5.5%) fulfilled the criteria for seropositivity based on the number of animals in the herd as described above. The geographical distribution of the 163 farms included in the study is shown in Figure 1. In these nine seropositive farms, 25 animals (of the 9,610 tested, 0.26%) were seropositive; all 25 seropositive animals were cows aged between 3.0 and 9.5 years, with a median of age of 5.0 years. A single cow was seropositive in one herd, two cows in six herds, and more than two (four and eight, respectively) in two farms. Herd size of the nine seropositive herds ranged from 36 to 138, with a median of 50 cows aged 2 years or more. Holstein was the predominant breed among the seropositive animals (14/25, 56%), and Jersey the second most predominant (9/25, 36%); the herd with the highest number of seropositive animals (eight among the 146 tested) was composed exclusively of Jerseys. The between-herd true seroprevalence calculated using a Se of 0.99 and a Sp of 0.98 was 3.6% (95% CI, 0.96–8.4%). The within-herd apparent seroprevalence in the nine seropositive herds ranged from 2.3 to 5.5% with a median of 3.6%, and the calculated within-herd true seroprevalence ranged from 2.8 to 8.3%, with a median of 4.9%. Due to the low apparent animal seroprevalence, it was not possible to calculate the true prevalence at animal level. Seropositive animals were present in 23 further farms that did not fulfill the criteria for seropositivity. Herd size of these farms ranged from 29 to 90 cows, with a median herd size of 54 cows. In 20 out of these 23 herds (87%), only one animal was seropositive, of which five were heifers; two cows were found to be seropositive in the remaining three herds.

### 3.3 Risk factor analysis on herd serostatus for paratuberculosis

The results of the univariable regression analysis regarding herd serostatus (positive or negative for PTB) are shown in Supplementary Table S1. Four variables exhibiting *p*-values below 0.2, were selected for inclusion in the subsequent multivariable regression analysis, i.e., “contact of lactating cows with animals from other herds during grazing,” “heifers sharing alpine pasture with animals from other herds,” “source of animals for purchase” and “herd size.” The results of the multivariable regression incorporating this variable set reaffirmed the significance of three of these four variables as explanatory factors for herd serostatus with *p*-values lower than 0.05, i.e., “contact of lactating cows with animals from other herds during grazing,” “heifers sharing alpine pasture with animals from other herds,” and “herd size” (Table 7A). The variables “herd size” and “contact of lactating cows with animals from other herds during grazing” emerged as significant positive contributors to the herd’s PTB serostatus, while “heifers sharing alpine pasture with animals from other herds “was identified as a significant negative contributor.

**Table 7:** Results of the multivariable logistic regression models assessing possible associations (A) between risk factors and herd serostatus (seropositive or seronegative for paratuberculosis) and (B) between risk factors and within-herd seroprevalence in 163 Swiss dairy herds (9 seropositive, 154 seronegative); statistically significant associations (*p* ≤ 0.05) are indicated in bold.

(A) Multivariable logistic regression model regarding risk factors for a positive herd serostatus					
Variable	Coefficient	OR^50^	SE^51^	95% CI^52^	*p* value
Intercept	−9.09	0	3.62	<0.000	0.012
**Contact of lactating cows with animals from other herds during grazing** Yes (vs. no)	3.6	36.8	1.47	2.05–658.54	**0.014**
**Heifers sharing alpine pasture with animals from other herds** Yes (vs. no)	−1.54	0.2	0.76	0.05–0.95	**0.042**
**Herd size (number of cows ≥ 2 years)**	1.83	6.2	0.88	1.11–35.05	**0.037**

(B) Multivariable logistic regression model regarding risk factors for within-herd prevalence				
Variable	Coefficient	SE^51^	95%CI^52^	*p* value
Intercept	0.96	0.24	0.49–1.43	<0.001
**Milk feeding** Powdered milk (vs. fresh milk)	**−0.68**	**0.24**	**−1.15–0.21**	**0.005**
Heifers sharing alpine pasture with other animal categories of their farm Yes (vs. no)	−0.24	0.17	−0.57–0.09	0.158
**Occurrence of diseases in lactating cows at the time of farm visit** Yes (vs. no)	**−0.33**	**0.14**	**−0.60–0.05**	**0.022**

^50^OR: Odd Ratio.

^51^SE: Standard Error.

^52^CI: Confidence Interval.

See also Tables 1–3 for definitions of the variables.

### 3.4 Risk factor analysis for within-herd seroprevalence

Three variables had *p*-values below 0.2 in the univariable regression analysis regarding within-herd seroprevalence, including “milk feeding,” “heifers sharing alpine pasture with other animal categories of the herd” and “occurrence of diseases in cows” (Supplementary Table S2). These variables were subsequently retained for inclusion in the multivariable regression model. Two of them, “milk feeding” and “occurrence of diseases in cows” remained significantly, albeit negatively associated with PTB seroprevalence (Table 7B).

### 3.5 Farmer’s knowledge about paratuberculosis

Questionnaire results on farmers’ knowledge about PTB are shown in Table 8. Only 24.5% of the participants had been aware of the existence of PTB prior to the study, the rest of them reported not having heard about the disease before and not being aware of clinical signs related to it. Among the herd managers who indicated having some knowledge of the disease, most recognized the two cardinal symptoms of chronic diarrhea and emaciation, while only few of them were aware of a reduction in milk yield and fertility. Only 35% of the farmers aware of the disease knew at least one infection pathway, whereby oral infection via feces and intrauterine infection were mentioned most often.

**Table 8:** Farmers’ knowledge about paratuberculosis (PTB) in 163 Swiss dairy herds (9 with seropositive and 154 with seronegative status), as assessed by interview.

	Total (%)	Seropositive farms (%)	Seronegative farms (%)
Farmers’ awareness of the existence of PTB			
Yes	40 (24.5)	6 (66.7)	34 (22.1)
No	123 (75.5)	3 (33.3)	120 (77.9)
Clinical signs of PTB listed by the farmers aware of the existence of PTB (multiple answers possible)^56^	n answers (%)		
Diarrhea	39 (97.5)	5 (83.3)	34 (100)
Emaciation	30 (75.0)	5 (83.3)	25 (73.5)
Reduction in milk yield	10 (25.0)	2 (33.3)	8 (23.5)
Reduction in fertility	2 (5.0)	0 (0)	2 (5.9)
Other clinical signs mentioned^57^	2 (5.0)	1 (16.6)	1 (2.9)
Transmission pathways of PTB listed by the farmers aware of the existence of PTB (multiple answers possible)^58^			
Direct fecal-oral transmission	16 (40.0)	3 (50.0)	13 (38.2)
Intrauterine infection	13 (32.5)	3 (50.0)	10 (29.4)
Transmission via MAP excretion into the milk of infected cows	7 (17.5)	1 (16.6)	6 (17.6)
Transmission via MAP excretion into the colostrum of infected cows	2 (5.0)	0 (0)	2 (5.9)
Pasture contamination by wild ruminants’ feces	1 (2.5)	0 (0)	1 (2.9)
Others transmission ways cited^59^	4 (10.0)	0 (0)	4 (11.8)
No transmission pathways known	18 (45.0)	3 (50.0)	15 (44.1)

^56^Clinical signs of PTB that were cited by the farmers when asked about the disease (open question).

^57^Other clinical signs cited by farmers as possibly associated with PTB (that are actually not directly related to the disease) included abortion and poor coat quality.

^58^Transmission pathways of PTB that were cited by the farmers when asked about the disease (open question).

^59^Other transmission pathways cited by the farmers (that are actually not directly related to PTB) included transmission via air, blood, placenta, and mucous membranes.

## 4 Discussion

The main aim of the present study was to estimate the national PTB herd and animal level prevalences in a large sample of Swiss dairy cattle herds, and to evaluate risk factors potentially associated with a positive herd serostatus and with the within-herd seroprevalence in positive herds. The apparent between-herd seroprevalence of 5.5% detected in this survey is distinctly lower than the apparent between-herd seroprevalence reported in previous studies, e.g., in a review of several studies conducted in European countries (38–68%) (60), or in a study conducted in Northern Italy (48% in Lombardy and 65% in Veneto (82)). At the individual animal level, the apparent seroprevalence of 0.53% is also lower than reported among adult cows in other Europeans countries, e.g., 2.6% in Lombardy and 4.0% in Veneto (82) or from 4.4 to 10.3% in Hungary (83). The median apparent within-herd seroprevalence was 3.6% in the nine seropositive herds. Previous prevalence records of MAP infection in Swiss dairy cattle are limited to few earlier studies (62–66). However, none of these studies provided reliable data for Switzerland due to small sample sizes or lack of representativity of the study population. Because participation in the present study was voluntary, a selection bias with farmers knowing or suspecting the presence of PTB in their herd and choosing not to participate for fear of possible consequences cannot be excluded. Although positive serological results had no regulatory consequences for the participating farms (this was confirmed in the mail sent to recruit participants), some farmers may have preferred not to be involved in a study on a notifiable disease. The recruitment of participating herds turned out to be more challenging than expected, although it was done in collaboration with the main breeding associations, with written confirmation by their boards that they recommended participation to this study on a disease of importance to the dairy industry. This eventually led to a number of participating herds lower than the calculated sample size. This suggests that (mandatory) sampling in a randomly selected subpopulation of dairy farms would be necessary for a really representative study on PTB in Switzerland. The exclusion of small farms in order to mitigate limited test accuracy through recruitment of herds with 25 cows or more may have further complicated the acquisition of participants for the study, given the relatively small average size of Swiss dairy herds. However, 40 animals or more were tested in most participating herds (121 out of 163, 74.2%). The farms included in the study were distributed in all agricultural zones of Switzerland (midland, hill, and mountain zones I-IV). This division into agricultural zones aims at representing the degree of difficulty in production and living conditions to be taken into consideration in the application of the Agriculture Act, e.g., some subsidies provided to the farmers depend on the zone where their farm is located. In the study population, a larger proportion of the farms (52.7%) was located in the midland zone compared to the proportion in the entire Swiss dairy population (32.5% of the herds). In contrast, the four mountain zones together were less represented in the study population in comparison with the general Swiss dairy farm distribution (32% vs. 52.2%). The distribution in the hill zone was the same in both populations (15.3%). This observation is likely due to the selection of farms with a minimal herd size of 25 dairy cows, as farms in the mountain zones tend to be smaller compared to the farms in the midland zone, with an average of 30.5 cows and 18.5 cows, respectively, in 2022 (84).

The observed seroprevalence is surprisingly low, despite the large number of samples collected in more than 160 herds. The serological analyses were conducted in duplicate in a certified laboratory (the Swiss reference laboratory for mycobacterial diseases in animals) with a commercial ELISA-kit, thus an unnoticed technical problem appears unplausible. One concern about using ELISA methods for the detection of positive herds is the possible misclassification of farms due to the low Se of serological tests in early stages of the infection, as antibodies are rarely present in sufficient quantities to be detected during the subclinical phase of PTB (21, 51). Based on the characteristics of the test used (Se = 58.2%, Sp = 99.3%), false negative results must be expected at the individual animal level. Given the contagious nature of PTB, it is not surprising that the odds of herd seropositivity should increase with herd size, which has been observed in numerous studies worldwide (14, 15, 66, 74, 85), including serological studies (12, 21, 22, 86). However, the higher probability of finding positive animals when more animals are tested in larger herds than in small herds must also be taken into consideration. This is the reason why we chose to sample and test all animals (from the age of 1 year old) in the participating herds, in order to increase the likelihood of detecting seropositive herds. Indeed, 146 (120 cows and 26 heifers) and 171 (138 cows and 33 heifers) animals were tested in the two farms with 8 and 4 seropositive animals, respectively, between 41 and 76 animals in the herds with two seropositive animals, and 36 in the smallest farm that was classified as positive with one seropositive cow. In order not to mitigate better Se with decreased Sp at the herd level due to the large number of tested animals, a cutoff (minimal number of seropositive animals needed to classify a herd as positive depending on the number of tested animals) was determined for each herd individually to ensure a Se and a Sp of 95% or more at herd level. Herd size, calculated based on the number of cows only in the models in order to include the herds with heifer rearing outside of the farm, remained significantly associated with a positive herd status, indicating that other factors than the number of tested animals alone contribute to the risk of being PTB positive in larger herds. These other factors may include, e.g., more animal purchase, larger groups of animals, or less control of management practices on large farms with hired employees than in small family farms.

The predominant breed in the participating farms was Holstein with 47.9% of the herds. These were mostly large herds, with a median herd size of 46.5 cows. In contrast, Swiss dairy farms are generally smaller with an average herd size of 23 cows (84). Although the Jersey breed has been reported to be more susceptible for PTB than other dairy breeds (85, 87, 88), breed was not identified as a risk factor in our analyses. The Jersey breed is not very common in Switzerland, as only 5,108 cows were registered with the breeding association in 2022 (71), which corresponded to 0.9% of the total dairy cow population in Switzerland (84). In our study, Jersey cattle was predominant in four herds (2.4%), one of which was composed exclusively of Jerseys. Nevertheless, 36% (9/25) of the seropositive animals in the positive herds were Jerseys, and the herd with the highest number of seropositive animals (8/146, 5.5%) was the one with Jersey cattle only. However, given the low number of observations, these results should not be overinterpreted.

Despite the precautions taken to minimize erroneous classification of herds, we know of at least one herd that was likely falsely classified as seronegative in the study as one cow (tested negative by ELISA in November 2021) later exhibited clinical signs of JD; according to the owner, the diagnosis was confirmed by examination of a fecal sample (method unknown). Thirty-five animals (24 cows and 11 heifers) had been tested in the herd, the positive cow was, to the best of our knowledge, the only animal that later exhibited signs of JD. There had already been a case of PTB in that herd in 2010 (confirmed by an unidentified laboratory analysis), but no other case had occurred since then until 2022. This underlines the limitations of serum ELISA at a single occasion even if all animals in the herd are tested. The use of more than one test, repeated over time to establish the disease status both of animals and herds, is recommended due to the well known diagnostic limitations for PTB (89). In the present study, every animal could be tested only once with an ELISA test due to practical constraints.

A secondary aim of the study was to identify the subpopulation at highest risk within seropositive herds, i.e., the one that should be preferably tested to determine the PTB status of a herd in the frame of a targeted sampling strategy. This objective could not be achieved given the low proportion of animals with a positive test result in any herd, as only one animal was seropositive in most of them (65.6%). Specific analyses to explore possible associations between individual serostatus and age or fertility (calving interval) of the tested animals did not reveal significant associations (data not shown). Thus, the results of this study suggest that the whole herd should be tested to establish its PTB status. A recommendation as to whether heifers between the age of 1 and 2 years should be tested to determine the PTB status of a herd cannot be made based on the present results, as none of the heifers that tested positive was in a herd that was eventually classified as seropositive. Nevertheless, positive FC results for MAP were found in a surprisingly high proportion of fecal samples from heifers in a previous study in Switzerland, suggesting that younger animals may play a role in the spread of MAP in infected herds and thus should be taken into account when trying to establish a herd status regarding PTB (66). Repeated testing would likely be the key to improve result accuracy (90). Testing of bulk tank milk using commercial ELISA methods is inexpensive and has been investigated for herd screening for JD, however, the results appear to be influenced by herd size and within-herd prevalence (91). Milk ELISA has been proven not to be sensitive enough to detect low prevalence herds (92, 93), thus it does not appear to be an adequate option to identify PTB infected Swiss dairy herds.

The strongest association between a risk factor and seropositive status of the herd was observed for “contact of lactating cows with cows from other herds during grazing” in the multivariable analysis. The lactating cows of three herds (one seropositive and two seronegative) shared grazing pastures around the farm (not alpine pastures) with cows from other herds. In two herds (herd size of 34 and 16 cows, respectively), the cows shared the pasture with cows from two other herds, in the third herd (44 cows) the cows shared the pasture with cows from nine other herds (data not shown). This practice appeared to be a potential risk factor for the herd of testing positive, however, this result must be interpreted with caution given the low number of farms with this characteristic. More data would be needed to further explore this potential risk factor. Nevertheless, keeping manure from cattle from other farms away from the herd by avoiding community or shared pasture has been recommended to decrease the risk of MAP introduction in dairy operations (94). Common pasturing has rather been suggested as a risk factor for young animals (calves and heifers) sharing pastures with adult cattle (95), which is in line with young animals being most susceptible to infection with MAP (6). In the present study, however, the variable “heifers sharing alpine pastures with animals from other farms” (mostly young animals, in 67.5% of the cases, but also with adult animals in 15.3%) was found to be associated with decreased odds of the herd testing seropositive, which was unexpected, especially as MAP shedding in heifers has been reported in a previous study in Switzerland (66). Common alpine pasturing of young animals is widespread in Switzerland (96) and the importance of alpine communal pasturing for the spread of other infectious diseases such as Bovine Virus Diarrhea (BVD) is well documented (97). In our study, heifers sharing alpine pastures with animals from other herds, was reported in 5 of 9 seropositive herds (55.6%, only with young animals) and 130 of 154 seronegative herds (84.4%, of which 68.2% with contact to young animals only and 16.2% to adult cattle), thus potential exposure to MAP may not have been the same in both groups. Although it may be postulated that the significant association may be related to low contact intensity due to extensive pasturing on alpine pastures or to the fact that PTB is not as highly contagious as BVD, these results must, again, be interpreted with caution given the low number of positive farms. Indeed, the odds ratios of all variables remaining in the final multivariable model exhibit wide confidence intervals (Table 7), indicating substantial uncertainty. Consequently, the interpretability of these risk factors is limited. This uncertainty primarily stems from the small number of seropositive herds and risk factors with limited discriminative power due to the presence of only a few observations within specific groups.

The identification of significant risk factors associated with high within-herd prevalence was rendered difficult by the low sensitivity of the ELISA test and the low within-herd prevalence, therefore the results of the analyses conducted to identify such factors must be interpreted with even greater caution than associations with the serostatus of the herds. It is, indeed, difficult to propose biologically rational explanations for the fact that the occurrence of diseases (mostly claw and udder problems were mentioned by the farmers) at the herd level in lactating cows would contribute to decreasing the risk of MAP spread in a positive farm. It must be pointed out that, e.g., relevant diseases in cows at the herd level were not observed in any of the 9 seropositive herds. Likewise, the calves were fed milk powder only (no fresh milk) in 22.2% of the seropositive herds (2/9) but in only 7.1% of the seronegative herds (11/154); this random repartition in our study population might explain, at least in part, why the feeding of milk powder, a common recommendation for PTB positive herds, appeared to be related to a low risk of MAP spreading.

In general, farmers’ knowledge about PTB was low, with more than 75.5% of the participants unaware of the very existence of the disease, of which 2.4% turned out having a seropositive herd. This suggest that signs of clinical disease would not be recognized as suspect of PTB by these farmers. After fulfilling the questionnaire, 17.2% of the participants could not exclude that they had had cases of PTB in their herd in the past, while most of them (74.8%) still stated that they had never had a case, although 2.4% of their herds were eventually classified as seropositive. Thirteen of the 163 farmers indicated that they had had at least one case (between one and five diseased animals per farm) in the 10 years prior to the study. While almost all 40 farmers who were aware of the existence of PTB were able to mention the cardinal symptoms of diarrhea (97.5%) and emaciation (75%), only 25% mentioned the reduction in milk yield and 5% also a reduction in fertility. The infection pathways were poorly understood by most, only 35% of the farmers aware of the disease were able to mention at least one infection pathway. The two mostly mentioned pathways were via feces (40%) and intrauterine infection (32.5%). Transmission through milk was rarely known (17.5%) and only 5% of the farmers were aware of possible transmission via colostrum. These results suggest that, despite previous studies in Switzerland (66, 98), PTB is still poorly known by Swiss farmers and further efforts to raise awareness are necessary. It is especially important to raise awareness about transmission through milk, due to the previously mentioned zoonotic potential of MAP, especially in a country with an important tradition in dairy production.

## 5 Conclusion

The results of the present study revealed a very low between-herd and within-herd prevalence of MAP seropositivity in Swiss dairy farms, even in large herds. This questions the true epidemiologic relevance of PTB in Switzerland. The low within-herd prevalence and the small size of Swiss dairy herds prevented the identification of a target population to be tested preferably to determine the PTB status of a dairy herd. In combination with the low performance, especially the low sensitivity, of serologic tests for the diagnosis of PTB, these results suggest that alternative methods (e.g., repeated PCR of environmental samples) may be more adequate and should be evaluated for Swiss dairy herds. Difficulties in recruiting farmers willing to participate in the study and their low level of awareness of PTB reveal knowledge gaps and poor understanding about infectious diseases, their transmission and the importance of biosecurity measures. Better information and education of Swiss dairy farmers about PTB and biosecurity in general should be a priority in the near future.

## Data availability statement

The original contributions presented in the study are included in the article/Supplementary material, further inquiries can be directed to the corresponding author.

## Ethics statement

The animal studies were approved by Amt für Veterinärwesen, 3000 Bern, Switzerland. The studies were conducted in accordance with the local legislation and institutional requirements. Written informed consent was obtained from the owners for the participation of their animals in this study.

## Author contributions

MO: Data curation, Formal analysis, Investigation, Methodology, Project administration, Validation, Writing – original draft, Writing – review & editing. IL: Data curation, Formal analysis, Investigation, Methodology, Writing – original draft, Writing – review & editing, Conceptualization, Funding acquisition, Resources, Software, Supervision, Validation. JW: Data curation, Formal analysis, Methodology, Software, Supervision, Writing – original draft, Writing – review & editing. SS: Conceptualization, Formal analysis, Funding acquisition, Investigation, Methodology, Supervision, Validation, Writing – original draft, Writing – review & editing. MS: Formal analysis, Investigation, Methodology, Supervision, Writing – original draft, Writing – review & editing. RSc: Investigation, Writing – original draft, Writing – review & editing. RSt: Conceptualization, Funding acquisition, Methodology, Supervision, Validation, Writing – original draft, Writing – review & editing. MM: Conceptualization, Data curation, Formal analysis, Funding acquisition, Investigation, Methodology, Project administration, Resources, Supervision, Validation, Visualization, Writing – original draft, Writing – review & editing.
