# Supplementary material for: Seroprevalence of Mycobacterium avium subsp. paratuberculosis in Swiss dairy herds and risk factors for a positive herd status and within-herd prevalence
Source: Front Vet Sci. 2024 Jun 28;11:1409694. doi: 10.3389/fvets.2024.1409694 (PMC11242548; doi:10.3389/fvets.2024.1409694)
Supplement: Supplementary file 1 [file table_1.pdf]

Supplementary Material

Seroprevalence of *Mycobacterium avium* subsp. *paratuberculosis* in Swiss dairy herds and risk factors for a positive herd status and within-herd prevalence

M. Ottardi[1], I. Lechner[2], J. Wang[2], S. Schmitt[3], M. Schneeberger[3], R.M. Schmid[1], R. Stephan[3], M. Meylan[1*]

[1]Clinic for Ruminants, Vetsuisse Faculty, University of Bern, CH-3012 Bern

[2]SAFOSO AG, CH-3097 Liebefeld

[3]Institute for Food Safety and Hygiene, Section of Veterinary Bacteriology, Vetsuisse Faculty, University of Zürich, CH-8057 Zürich

*** Correspondence:**
Mireille Meylan
mireille.meylan@unibe.ch

1 Supplementary Table S1

Table S1. Results of the univariable logistic regression model assessing possible associations between risk factors and herd serostatus (seropositive or seronegative for paratuberculosis) in 163 Swiss dairy herds (9 seropositive, 154 seronegative); variables with p<0.2 are indicated in bold and were carried forward for the multivariable regression model

Variable	Coefficient	OR[1]	SE[2]	95% CI[3]	p value
Type(s) of production on the farm<br>Dairy with fattening of own or purchased animals<br>(vs. exclusively dairy)	1.13	3.1	1.14	0.33-28.77	0.323
Presence of a bull in the herd<br>Purchased<br>(vs. no or born on the farm)	-0.4	0.7	0.82	0.13-3.35	0.627
Origin of the farm's water supply<br>Private well<br>(vs. communal water only)	0.12	1.1	0.69	0.29-4.35	0.865
Contact of heifers with animals from other herds during the grazing period<br>Yes<br>(vs. no)	-0.16	0.9	0.73	0.20-3.53	0.824
**Contact of lactating cows with animals from other herds during grazing**<br>Yes<br>(vs. no)	2.25	9.5	1.28	0.78-116.15	**0.078**
Contact of dry cows with animals from other herds during the grazing period<br>Yes<br>(vs. no)	1.32	3.7	1.15	0.39-35.76	0.254
Pre-weaned calves sharing alpine pasture with animals from other herds<br>Yes<br>(vs. no)	-13.74	<0.001	1696.73	0-Inf	0.994

Post-weaned calves sharing alpine pasture with animals from other herds<br>Yes<br>(vs. no)	-15.88	<0.001	1390.63	0-Inf	0.991
**Heifers sharing alpine pasture with animals from other herds**<br>Yes<br>(vs. no)	-1.47	0.2	0.71	0.06-0.92	**0.038**
Lactating cows sharing alpine pasture with animals from other herds<br>Yes<br>(vs. no)	-0.58	0.6	1.08	0.07-4.68	0.595
Dry cows sharing alpine pasture with animals from other herds<br>Yes<br>(vs. no)	-15.88	<0.001	1390.63	0-Inf	0.991
Participation to cattle shows<br>Yes<br>(vs. no)	-0.22	0.8	0.73	0.19-3.34	0.765
Purchase of breeding animals<br>Yes<br>(vs. no)	0.01	1	0.73	0.24- 4.20	0.989
**Source of animals for purchase**<br>Purchase from ≥1 farm<br>(vs. no purchase)	1.2	3.3	0.82	0.67-16.51	**0.142**
Information requested about the source farms prior to purchase<br>Yes<br>(vs. no)	-0.82	0.4	1.12	0.05-3.95	0.462
Heifers raised on rearing farm<br>Yes<br>(vs. no)	-0.58	0.6	0.82	0.11-2.79	0.48
**Herd size (all cows ≥2 years)**	1.6	5	0.8	1.03-23.89	**0.046**

[1]OR: Odd Ratio
[2]SE: Standard Error
[3]CI: Confidence Interval
See also Tables I and II for definitions of the variables
