# Supplementary material for: Seroprevalence of Mycobacterium avium subsp. paratuberculosis in Swiss dairy herds and risk factors for a positive herd status and within-herd prevalence
Source: Front Vet Sci. 2024 Jun 28;11:1409694. doi: 10.3389/fvets.2024.1409694 (PMC11242548; doi:10.3389/fvets.2024.1409694)
Supplement: Supplementary file 2 [file table_2.pdf]

Supplementary Material

Seroprevalence of *Mycobacterium avium* subsp. *paratuberculosis* in Swiss dairy herds and risk factors for a positive herd status and within-herd prevalence

M. Ottardi[1], I. Lechner[2], J. Wang[2], S. Schmitt[3], M. Schneeberger[3], R.M. Schmid[1], R. Stephan[3], M. Meylan[1]*

[1]Clinic for Ruminants, Vetsuisse Faculty, University of Bern, CH-3012 Bern

[2]SAFOSO AG, CH-3097 Liebefeld

[3]Institute for Food Safety and Hygiene, Section of Veterinary Bacteriology, Vetsuisse Faculty, University of Zürich, CH-8057 Zürich

*** Correspondence:**
Mireille Meylan
mireille.meylan@unibe.ch

1 Supplementary Table S2

Table S2. Results of the univariable logistic regression model assessing possible associations between risk factors and within-herd seroprevalence in 163 Swiss dairy herds (9 seropositive, 154 seronegative); variables with $p<0.2$ are indicated in bold and were carried forward for the multivariable regression model

Variable	Coefficient	SE[1]	95% CI[2]	p value
Presence of other ruminants on the farm				
Yes	0.08	0.14	-0.20-0.36	0.58
(vs. no)				
Presence of pigs on the farm				
≥ 20 pigs	0.01	0.19	-0.37-0.39	0.95
< 20 pigs	-0.21	0.25	-0.71-0.29	0.42
(vs. none)				
Contact with other cows during calving				
Intensive contact (group calving box)	0.03	0.15	-0.25-0.32	0.80
Low contact intensity	-0.003	0.19	-0.38-0.37	0.98
(vs. only individual calving)				
Bedding management for calving				
Same bedding used for several calvings	0.04	0.28	-0.14-0.95	0.41
Pen or stall freshly bedded before each calving	-0.027	0.17	-0.61-0.07	0.26
(vs. change of bedding after each calving)				
Gloves for the care of newborn calves				
Yes	0.2	0.35	-0.49-0.90	0.57
(vs. no)				
Use of the calving pen as sick pen	0.02	0.05	-0.08-0.12	0.68
Always	0.08	0.18	-0.27-0.43	0.65
Often	-0.11	0.23	-0.58-0.35	0.62
Rarely	0.05	0.19	-0.34-0.44	0.80
(vs. never)				
Drinking of colostrum from the dam's udder				
Yes	-0.14	0.13	-0.40-0.12	0.30
(vs. no)				
Housing of the young animals				
Neonates (first 2-3 weeks of life)				

Group pen	0.02	0.13	-0.24-0.28	0.89
(vs. individual hutch)				
Pre-weaned calves				
Not on the farm (rearing farm)	0.2	0.49	-0.77-1.18	0.68
Group pen	-0.02	0.65	-1.28-1.28	1
(vs. individual hutch)				
Post-weaned calves				
Not on the farm (rearing farm)	0.11	0.18	-0.24-0.47	0.52
(vs. group pen)				
Risk of fecal contamination from the cows to the calves' environment				
Neonates (first 2-3 weeks of life)				
High risk (direct contact in the barn)	-0.25	0.52	-1.27-0.77	0.62
Low risk (indirect contact through equipment and/or boots)	-0.39	0.43	-1.24-0.44	0.35
(vs. no risk)				
Pre-weaned calves				
High risk (direct contact in the barn)	-0.01	0.47	-0.94-0.92	0.97
Moderate risk (direct contact on pasture)	-0.38	0.6	-1.57-0.8	0.52
Low risk (indirect contact through equipment and/or boots)	-0.2	0.35	-0.9-0.49	0.57
(vs. no risk)				
Post-weaned calves				
High risk (direct contact in the barn)	-<0.001	0.41	-0.81-0.81	1
Moderate risk (direct contact on pasture)	0.21	0.24	-0.26-0.68	0.38
Low risk (indirect contact through equipment and/or boots)	0.25	0.27	-0.28-0.79	0.35
(vs. no risk)				
Frequency of manure removal				
In the neonates' area				
<1x/day	0.22	0.18	-0.13-0.59	0.22
(vs. 1-2x/day)				
In the pre-weaned calves' barn				
<1x/day	-0.54	0.5	-1.52-0.44	0.28
1-2x/day	-0.77	0.52	-1.79-0.25	0.24
(vs. >3x/day)				
In the post-weaned calves' barn				
<1x/day	0.02	0.25	-0.46-0.51	0.92
1-2x/day)	-0.08	0.26	-0.59-0.42	0.74
(vs. >3x/day)				
In the heifers' barn				
<1x/day	0.17	0.22	-0.26-0.62	0.43
1-2x/day)	0.07	0.22	-0.36-0.52	0.72
(vs. >3x/day)				
In the lactating cows' barn				
<1x/day	-0.13	0.25	-0.64-0.36	0.59
1-2x/day)	0.25	0.15	-0.04-0.55	0.10
(vs. >3x/day)				
In the dry cows' barn				
<1x/day	0.09	0.17	-0.24-0.43	0.58
1-2x/day)	0.07	0.15	-0.22-0.37	0.61
(vs. >3x/day)				
Cleaning management in the calf area	0.02	0.06	-0.10-0.14	0.75
In the neonates' area				
No disinfection, cleaning or washing	0.07	0.24	-0.4-0.54	0.77
Cleaning or washing	-0.17	0.33	-0.83-0.48	0.60
Disinfection 1-4x/year	0.02	0.23	-0.44-0.48	0.93
(vs. regular disinfection[3])				
In the pre-weaned calves' barn				
No disinfection, cleaning or washing	-0.10	0.29	-0.68-0.47	0.73
Cleaning or washing	-0.30	0.67	-1.62-1	0.64
Disinfection 1-4x/year	-0.19	0.33	-0.84-0.45	0.56
(vs. regular disinfection[3])				

In the post-weaned calves' barn				
No disinfection, cleaning or washing	0.21	0.86	-1.47-1.89	0.81
Cleaning or washing	<0.001	1.21	-2.37-2.37	1
Disinfection 1-4x/year	<0.001	0.90	-1.77-1.77	1
(vs. regular disinfection[3])				
**Milk feeding**				
Powdered milk (vs. fresh milk)	-0.63	0.24	-1.10--0.15	**0.01**
Leftovers from the cows' feed				
Given to post-weaned calves				
Yes (vs. no)	-0.21	0.23	-0.66-0.23	0.35
Given to heifers				
Yes (vs. no)	0.04	0.16	-0.28-0.36	0.81
Possible fecal contamination of the calves' feed with feces of older animals Yes (vs. no)	-0.23	0.29	-0.80-0.34	0.43
Possible contamination of the calves' water with feces of older animals Yes (vs. no)	-0.20	0.5	-1.17-0.78	0.69
Runoff from the manure pile in the environment Yes (vs. no)	0.18	0.15	-0.11-0.46	0.23
Use of manure equipment for feed transport Yes (vs. no)	-0.20	0.39	-0.96-0.56	0.60
Use of manure and/or slurry for fertilization				
For pastures, including calf pastures	0.21	0.4	-0.55-0.97	0.60
For cows' and heifers' pastures	0.18	0.4	-0.6-0.97	0.64
(vs. for crop land and/or hay meadows)				
**Heifers sharing alpine pastures with other animal categories from their farm** Yes (vs. no)	-0.24	0.17	-0.58-0.10	**0.17**
Lactating cows sharing alpine pastures with other animal categories from their farm Yes (vs. no)	-0.2	0.33	-0.85-0.44	0.54
Dry cows sharing alpine pastures with other animal categories from their farm Yes (vs. no)	-0.22	0.23	-0.67-0.24	0.35
Occurrence of diseases in calves at the time of farm visit Yes (vs. no)	-0.18	0.16	-0.49-0.13	0.25
Occurrence of diseases in heifers at the time of farm visit Yes (vs. no)	0.23	0.37	-0.50-0.97	0.53
**Occurrence of diseases in lactating cows at the time of farm visit** Yes (vs. no)	-0.28	0.14	-0.56-0.00	**0.05**

[1]SE: Standard Error
[2]CI: Confidence Interval
[3]Regular disinfection: after each use (individual igloo) or each group
See also Tables I and III for definitions of the variables
